# Whole and Isolated Protein Fractions Differentially Affect Gastrointestinal Integrity Markers in C57Bl/6 Mice Fed Diets with a Moderate-Fat Content

**DOI:** 10.3390/nu13041251

**Published:** 2021-04-10

**Authors:** Tara R. Price, Sangeetha A. Baskaran, Kristin L. Moncada, Yasushi Minamoto, Cory Klemashevich, Arul Jayuraman, Jan S. Sucholdoski, Luis O. Tedeschi, Jörg M. Steiner, Suresh D. Pillai, Rosemary L. Walzem

**Affiliations:** 1 Department of Nutrition, Texas A&M University, College Station, TX 77843, USA; trprice@wisc.edu; 2 Department of Poultry Science, Texas A&M University, College Station, TX 77843, USA; girsan81@gmail.com (S.A.B.); Kristin.Moncada@pilgrims.com (K.L.M.); 3 Gastrointestinal Laboratory, Dept. Small Animal Clinical Sciences, College of Veterinary Medicine and Biomedical Sciences, Texas A&M University, College Station, TX 77843, USA; Yasushi.Minamoto@gmail.com (Y.M.); jsuchodolski@cvm.tamu.edu (J.S.S.); jsteiner@cvm.tamu.edu (J.M.S.); 4 Department of Chemical Engineering, Texas A&M University, College Station, TX 77843, USA; klem24@tamu.edu (C.K.); Arulj@tamu.edu (A.J.); 5 Department of Animal Science, Texas A&M University, College Station, TX 77843, USA; luis.tedeschi@tamu.edu; 6 Graduate Faculty of Nutrition, Texas A&M University, College Station, TX 77843, USA; s-pillai@tamu.edu; 7 Department of Food Science and Technology, Texas A&M University, College Station, TX 77843, USA

**Keywords:** bodyweight, gastrointestinal permeability, saturated fatty acids, dairy proteins, myeloperoxidase, intestinal health, isolated soy protein

## Abstract

Various proteins or protein fractions reportedly positively affect gastrointestinal integrity and inflammation in diets providing >45% energy as fat. This study tested whether benefits were seen in diets providing 30% of energy as fat. Purified diets (PD) with isolated soy protein (ISP), dried whole milk powder (DWMP), milk fat globule membrane (MFGM), or milk protein concentrate (MPC) as protein sources were fed to C57BL/6J mice (*n =* 15/diet group) for 13 weeks. MFGM-fed mice were heaviest (*p* < 0.005) but remained within breeder norms. Growth rates and gut motility were similar for all PD-fed mice. FITC-dextran assessed gut permeability was lowest in DWMP and MFGM (*p* = 0.054); overall, plasma endotoxin and unprovoked circulating cytokines indicated a non-inflammatory state for all PD-fed mice. Despite differences in cecal butyrate and intestinal gene expression, all PDs supported gastrointestinal health. Whole milk provided more positive effects compared to its fractions. However, ISP-fed mice showed a >370%, (*p* < 0.006) increase in colonic myeloperoxidase activity indicative of tissue neutrophil infiltration. Surprisingly, FITC-dextran and endotoxin outcomes were many folds better in PD-fed mice than mice (strain, vendor, age and sex matched) fed a “chow-type” nutritionally adequate non-PD. Additional variables within a diet’s matrix appear to affect routine indicators or gastrointestinal health.

## 1. Introduction

Several factors influence gut health, some of which are host-based and others that are related to the intestinal microbiome. Nutrient composition and the food matrix that delivers those nutrients influence both intestinal tissue and microbiota physiology and, ultimately, host health. Several studies have evaluated associations between dietary components and intestinal integrity [1,2,3,4,5]. Diet-microbiome interactions produce metabolites critical to intestinal epithelial health and can directly contribute to metabolic phenotypes [6,7,8]. Obesity, a leading cause of metabolic disease, increases the complexity of interactions among host, diet and microbiota. Obesity appears to act in part by promoting increased gut permeability that promotes inflammation through the passage of microbial inflammogens that lead to increases in plasma cytokines [9,10,11].

Dairy milk, an animal protein source recommended as part of USDA’s “MyPlate” [12], is a complex food product that provides vitamins and minerals, as well as oligosaccharides, protein, and lipids. Human clinical and epidemiological studies established associations between dairy intake and reduced colorectal cancer risk, as well as improved body weight regulation [13,14,15]. Initially, such associations were ascribed to calcium provided by dairy foods [14,16]; however, specifically designed animal studies provided evidence that dairy protein and lipid components served active roles in those beneficial effects [17,18,19]. Milk digestion gives rise to novel bioactive and protein-lipid moieties [20,21,22,23]. Previous studies using dairy protein sources showed non-fat dry milk (NFDM) reduced body fat gain and inflammatory responses compared to isolated soy protein in a mouse model in which dietary fat provided 45% of energy (EN) [17,24]. In a LPS challenge model, a high-fat diet containing milk fat globule membrane (MFGM) protected against gut leakiness and reduced pro-inflammatory cytokines [19]. MFGM reduced the incidence of aberrant crypt foci in Fisher-344 rats, despite no change in mucosal gene expression associated with cancer risk [18]. 

Many studies that compare health impacts of dairy proteins to those of soy often do so in the context of feeding a high-fat diet [18,19,25,26]. For example, milk-derived saturated fats were shown to promote colitis and microbial dysbiosis in an IL-10^−/−^ mouse model fed a high-fat diet that provided 37% of calories as fat [25]. However, no studies have compared the effects of whole (i.e., “full-fat” or 4% fat by weight) dairy milk or its fractions to those of isolated soy protein within the context of moderate-fat diets formulated to provide 30% EN as fat, 20% EN as protein and 50% EN as carbohydrate. Such a distribution is similar to that found in standard mouse breeder chow and promulgated by the Dietary Guidelines for Americans [27,28]. The potential for whole milk products to influence body weight gain, gut health, and inflammatory responses is not well-studied. We sought to determine the effects of whole milk or its fractions compared to a plant-based protein source in diets containing similar caloric densities. Full-fat dairy, as dried whole milk powder (DWMP), or its fractions, milk protein concentrate (MPC) and MFGM, were evaluated along with isolated soy protein (ISP) serving as a plant-based protein comparator.

## 2. Materials and Methods

### 2.1. Purified Diet Rationale and Composition

Purified diets were isocaloric and isonitrogenous with protein provided as either ISP, DWMP, MPC, and MFGM (Table 1, Supplementary Tables S1–S3). Macronutrient values were provided by the vendor (ENVIGO/Harlan Laboratories, Indianapolis, IN, USA). Mineral content of composed diets was measured using ICP-MS following wet ashing in nitric acid (Texas A&M Soil and Forage Testing Laboratory, College Station, TX, USA). Dietary amino acid composition was determined by AOAC Official Method 982.30 E(a,b,c), chp. 45.3.05, 2006 (Agricultural Experiment Station Chemical Laboratories, University of Missouri, Columbia, MO, USA). Dietary fatty acid profiles were determined using gas chromatography as previously described but both standard GLC-674 and GLC-68d (NuChek Prep, Elysian, MN, USA) were used to identify fatty acids with chain lengths of 10 or less [29]. ISP and MPC contained lard as the primary lipid source, with saturated fats averaging 34% of fatty acids (Supplementary Table S3) and cholesterol contents of 95 and 139 ppm, respectively. DWMP and MFGM contained dairy fat as the primary lipid source with saturated fats equaling 56% of total fatty acids and cholesterol contents of 378 and 74 ppm, respectively. The MFGM protein was prepared according to [19] by Dr. Jimenez-Flores, and contains membrane proteins and intrinsic [30] phospholipids. Diets were formulated as individual treatments with ISP and DWMP formulations following those of [17,24] in order to assess outcomes in a lower fat context. Similarly, the MFGM diet followed that of [19] with a lower fat content. Milk protein concentrate is prepared from skimmed milk but retains whey:casein of whole milk; at the 85% protein concentration used in the present diet formulation (MPC85, Erie Foods International, Inc. Erie, IL, USA) lactose, non-casein bound milk salts and most fats are greatly reduced compared to whole milk powder [31] allowing a milk protein comparison to ISP in the same non-dairy lipid context through the use of lard as added dietary fat.

### 2.2. Purified Diets—Animal Growth

The Institutional Animal Care and Use Committee (IACUC) at Texas A&M University approved all animal studies (IACUC 2016-0084). Weanling (21-day-old) C57Bl6 male mice (Jackson Labs, Bar Harbor, ME, USA) were housed individually using a 12-h light/dark cycle (6 am on/6 pm off). Mice were provided with a nutritionally adequate closed-formula, non-purified diet (“Chow”, Teklad Rodent Diet #8604, Harlan Laboratories, Indianapolis, IN, USA) for seven days following receipt to adapt them to local environmental conditions and measure growth rates. On day seven of each trial, four groups of five animals each were formed to provide similar starting body weights and growth rates. Group assignment to a purified diet (PD) treatment was random. Animals had free access to diets during each 13-week growth trial. Following CO_2_ asphyxiation, mice were exsanguinated by cardiac puncture. Whole blood was collected into heparinized tubes and plasma separated within two hours following centrifugation. Liver, thymus, spleen, gastrocnemius muscle, and retroperitoneal fat pads were collected, weighed, and stored at −80 °C. Following gastrointestinal tract excision and length measurement, it was divided into the proximal and distal small intestine, cecum, and ascending and descending colon. Tissue sections were further divided for histology, enzyme assays and gene expression analysis. Cecal contents, retained for measurement of short-chain fatty acids and indole concentrations, were flash-frozen in liquid nitrogen and stored at −80 °C.

### 2.3. Plasma Endotoxin Assays

Bacterial endotoxin transfer into circulation was measured in plasma by the limulus amebocyte lysate (LAL) assay with Glucashield^®^ buffer (Associates of Cape Cod, Inc. (ACCI), East Falmouth, MA, USA). Plasma, diluted 1:10 in endotoxin-free water, was plated into a 96-well microplate with an equal volume of diluted lysate and incubated for 74 min at 37 °C. The reaction was stopped by adding 50% acetic acid, and the absorbance read immediately at 405 nm. Sample concentrations were determined from a standard curve created from Control Standard Endotoxin (ACCI). In cohorts 1 and 2, the LAL assay provided limited distinction between dietary groups, which led us to question whether other methods may be more revealing. Therefore, in a third cohort of mice (*n =* 5), we pursued avenues to interrogate alterations in in vivo permeability, intestinal enzyme activities and gene expression. 

### 2.4. In Vivo Gastrointestinal Permeability and Motility 

At week 12 of experimental diet feeding, all mice were gavaged with 0.2 mL of 5% Evans blue dye suspended in 5% gum Arabic to assess gut motility [32]. Whole gut transit equaled time to first blue feces. In a subset of five mice per diet group, gastrointestinal permeability (GI-P) was assessed by the inclusion of 4kDa FITC-dextran (600 mg/kg BW, Sigma-Aldrich, St. Louis, MO, USA) in the 5% Evans blue dye/5% gum Arabic solution [8,33,34]. Gut permeability equaled net plasma fluorescence concentration four hours post-dosing following zero-hour value subtraction as measured in a microplate reader (Biotek Instruments, Winooski, VT, USA) with excitation at 485 nm and emission at 535 nm. Plasma FITC-dextran concentrations were determined from a 5-point standard curve.

### 2.5. Plasma Cytokines and IgA

The inflammatory cytokines TNF-α, IL-12p70, IL-6, IFN-γ, MIP-2, IL-1β and anti-inflammatory cytokine IL-10 were measured in plasma using a multiplex magnetic bead-based assay (Millipore, Burlington, MA, USA). Mouse Serum Matrix (#MXMSM, Millipore, Burlington, MA, USA) was used as a sample diluent and as a reagent blank prior to calculations. Plates were read on a Luminex 200 xMAP system and a 6-point standard curve generated by xPONENT analysis software (version 3.1, Luminex Corp., Austin, TX, USA). Sample plasma concentrations were calculated based on the standard curve. Validation of individual runs were assessed with manufacturer-supplied quality control samples. Plasma TNF-α concentrations were considered physiologically significant at 3.0 pg/mL or greater [35,36,37]. Likewise, IL-6 concentrations greater than 3.5 pg/mL [35,36,37] and IL-1β greater than 10.0 pg/mL [38,39] were considered physiologically relevant.

IgA concentrations were measured with a commercially available ELISA (E99-103, Bethyl Laboratories, Montgomery, TX, USA) following manufacturer instructions.

### 2.6. Intestinal Enzyme Assays

Intestinal enzymes were measured in whole tissue sections (*n =* 5/diet treatment) that had been flash-frozen in liquid nitrogen and stored at −80 °C until assayed. Myeloperoxidase (MPO) activity in whole descending colon homogenates (1:4 tissue:assay buffer) were measured using a commercially available fluorometric MPO assay kit (ab111749, Abcam Inc., Cambridge, MA, USA) following manufacturer instructions for one hour kinetic measurements in black microplates (Ex/Em 485/520, Synergy2 microplate reader using Gen5 Software (Biotek Instruments, Winooski, VT, USA)). Colonic MPO activity was calculated from fluorescein generated in MPO standards (1.0 unit MPO activity = MPO required to generate 1.0 umol fluorescein/minute from oxidized aminophenyl fluorescein). 

Intestinal alkaline phosphatase (ALP) protein concentrations in whole tissue homogenates (*n =* 6/diet treatment, 400 µL lysis buffer/50 mg tissue) of the small intestinal and colonic sections were determined using the SensoLyte pNPP ALP assay kit (AS-72146, Anaspec, Fremont, CA, USA) following centrifugation. Cleared tissue supernatants from small intestinal samples were diluted 1:100 or 1:200, while those from the colon were diluted 1:50 with lysis buffer prior to assay. Standards and diluted samples were incubated with pNPP reaction mixture for 25 min and the absorbance read at 405 nm. Sample concentrations were calculated based on a 7-point standard curve.

### 2.7. Histology

Freshly harvested distal small intestinal tissue was immediately submersion fixed (Z-fix, Anatech, Ltd., Battle Creek, MI, USA) for 24–36 h, rinsed with PBS, and stored in 70% ethanol prior to embedding in paraffin and staining with hematoxylin and eosin (H&E; TAMU College of Veterinary Medicine Histology Lab, College Station, TX, USA). Immunohistochemistry staining for CD3, a T-cell co-receptor, was performed by the TAMU Histopathology Laboratory. Stained sections were scanned at 20× magnification on a Hamamatsu C9600-12 slide scanner (Hamamatsu Photonics, Hamamatsu City, Japan). NDP.view2 software (Hamamatsu Photonics) was used for manual analyses of scanned images for villous height and width, crypt depth and width and smooth muscle thickness. Average values were calculated from a minimum of 20 measurements per morphological characteristic for each sample. Subsequent data analyses used average values for each sample.

### 2.8. Gene Expression 

Gene expression was analyzed using real-time reverse transcriptase (RT-qPCR) and the comparative C_T_ method (ΔΔC_T_) for relative quantitation of diet groups (Supplementary Table S4). Frozen intact proximal small intestine or retroperitoneal adipose tissue was homogenized in TRizol (ThermoFisher, Waltham, MA, USA) with a tissue homogenizer and total RNA was extracted using RNeasy mini kit (#74104, Qiagen, Germantown, MD, USA). Genomic DNA was removed from total RNA by DNase treatment using Ambion TURBO DNA-free™ (#AM1907, ThermoFisher, Waltham, MA, USA). The quantity and quality of RNA were evaluated using an Eppendorf Biophotometer (Eppendorf, Hamburg, Germany); a minimum 1.8 260/280 absorbance ratio was considered acceptable. Reverse transcription of normalized RNA into cDNA used the High Capacity cDNA Reverse Transcription Kit protocol (Applied Biosystems (ABI), Foster City, CA, USA). cDNA was used as a template for RT-qPCR amplification of the target gene (Supplementary Table S4). RT-qPCR reactions were performed using the 7500 Real-Time fast PCR system with SYBR Green reagents (ABI). The comparative critical threshold (C_T_) method determined the relative mRNA levels. β-actin (ACTB) was used as an endogenous control (Supplementary Figures S1–S3). Intestinal samples from ISP-fed mice served as the calibrator to obtain relative quantitative expression of genes for DWMP, MPC and MFGM-fed intestinal tissue samples. In this method, average ΔΔC_T_ of each diet group is calculated to produce a singular value which is then used in the 2^− ΔΔCT^ formula. The comparator group, ISP, is set to a fold change of one. Subsequently, fold changes of DWMP, MPC, and MFGM are calculated relative to ISP. Because average values for both the comparator and test groups, a singular value without standard errors is obtained. Gene fold differences of 2.0 or greater, or its reciprocal 0.5 or less, were considered significant. 

### 2.9. Cecal Metabolite Content

Stable isotope dilution gas chromatography was used to quantify short-chain fatty acids (SCFA) and branched-chain fatty acids (BCFA) [40]. Acetic acid, propionic acid, butyric acid, isovaleric acid, isobutyric acid, and valeric acid were measured. Weighed cecal samples were diluted 1:5 in 2N HCl and homogenized for 30 min at room temperature, centrifuged to pellet solids prior to supernatant harvest. 500 µL of supernatant was mixed with 10 µL internal standard and extracted using a C18 solid phase extraction column. Samples were derivatized with *N*-tert-butyldimethylsilyl-*N*-methyltrifluoroacetamide (MTBSTFA) prior to separation and quantification by GC-MS [40]. The ratio of area under the curve (AUC) to the internal standard curve was used to quantify SCFA/BCFA concentrations. Concentrations were determined based on grams of lyophilized cecal content used for extraction. 

Fresh-frozen cecal samples were homogenized in 1:3 chloroform:methanol, centrifuged and the supernatant was collected for indole extraction [6]. Following distilled water addition, samples were briefly centrifuged to separate the methanol and chloroform phases. LC-MS was performed on reconstituted samples following the protocol published by Jin, et al. [41].

### 2.10. Chow-Fed Reference Mice 

Chow-fed mice often serve as a “gold standard” for laboratory mouse growth and health. An extensive literature search for defined clinical values for GI-P of healthy C57Bl/6 mice failed. Within laboratory reference values for FITC-dextran and endotoxin estimates of GI-P were determined in an age-matched cohort of mice (*n =* 10). Mice were 16-week-old C57Bl/6 male mice (Jackson Laboratories, Bar Harbor, ME, USA). Mice were housed at TAMU facilities with standard 12-h light/dark cycles and maintained on Teklad Rodent Diet (Product # 8604, 4% fat by weight, Harlan, Madison, WI, USA; Supplementary Tables S2 and S3) for two weeks to acclimate to local conditions prior to use in relevant assays. 

### 2.11. Statistical Analyses

The effect of diet treatments on the variables of interest was determined with PROC MIXED of SAS (SAS Inst., Cary, NC, USA), assuming initial body weight as covariate and cohorts (1, 2 or 3) as a random factor to account for the heterogeneity in the covariance structure. A preliminary analysis indicated that initial body weight was not different among treatments (*p*-value = 0.183) and could be used as a covariate. The restricted maximum likelihood was used to estimate the covariate parameters. Because degrees of freedom were different among treatments, the Kenward–Roger method was used to compute the denominator degrees of freedom. The least-square means method was used for multiple comparisons of treatment means after adjusting the p-value for Tukey. Significance was assumed when *p*-value was equal to or less than 0.05. For parameters lacking diet-cohort interaction (i.e., measurement of serum parameters and gene expression data), one-way ANOVA and Tukey’s HSD were used. Data are reported as means ± standard error of the mean unless otherwise noted.

## 3. Results

### 3.1. Animal Growth and Development 

Final body weights differed after 13 weeks on experimental diets (Table 2, *p* < 0.002). Across all cohorts (*n =* 15/dietary treatment), MFGM-fed mice were heaviest, having the highest total weight gain and % weight gain (Table 2, *p* < 0.002). Feed intake was greatest with DWMP, and was significantly higher than MPC (Table 2, *p* < 0.004). 

Organ weights and fat pads trended similarly to bodyweight outcomes, with MFGM being mostly associated with the heaviest organ weights (Table 3). Liver and fat pad weights were significantly heavier for mice fed MFGM, compared to those fed either the DWMP (+21%) or the MPC (+24%) diet.

At the end of the experiment, DWMP fed mice had the heaviest cecum, which was significant compared to the cecal weight of MFGM fed mice. Organ weights expressed as % of final body weight for gastrocnemius muscle, spleen and thymus were similar for all groups (Supplementary Table S5). Fractional weights of liver and retroperitoneal fat pads (RPFP) decreased in order of MFGM > ISP > MPC > DWMP with the difference between MFGM and DWMP being significant (*p* < 0.005 and *p* = 0.024 for liver and RPFP, respectively). Dietary protein source did not significantly alter the length of either the small intestine (*p* = 0.77) or colon (*p* = 0.40).

### 3.2. In Vivo Gastrointestinal Permeability and Motility

Mice fed the DWMP-based diet had the highest intestinal integrity as indicated by the lowest FITC-dextran concentration in peripheral blood, with values close to statistical significance compared to ISP (Table 4, *p* = 0.054). Mice fed ISP and MPC diets had the highest plasma concentration of FITC-dextran, while those fed DWMP and MFGM had lower levels of FITC-dextran than mice fed ISP. We found low and similar plasma endotoxin concentrations in mice from all PD groups (Table 4). 

### 3.3. Systemic Inflammatory Markers

The macrophage marker CD11d (*ITGAD*) in adipose tissue did not differ among dietary treatments. The value for ISP was set to 1.0, and fold changes of the dairy protein-based diets were DWMP = 1.38, MPC = 1.61, MFGM = 0.83. Plasma cytokine concentrations were low (Table 4). Notably, despite all values being technically valid by assay standards, many values were near or below the limit of detection. Table 4 shows that plasma IgA concentration was highest in MFGM-fed mice, being 35% higher than that in DWMP- or MPC-fed mice, and 75% increased over ISP-fed mice (*p* < 0.000). MPC-fed mice had the highest TNF-α concentrations, a 3.76-fold increase over MFGM-fed mice (18.4 pg/mL vs. 4.84 pg/mL, *p* = 0.007). Plasma TNF-α concentrations in ISP- and DWMP-fed mice were below the limit of detection for the assay. 

### 3.4. Intestinal Enzyme Assays

Myeloperoxidase activity, a marker of neutrophil infiltration, was similar among mice fed dairy protein-based diets (Table 4, *p* = 0.873). However, MPO activity increased >370% in the distal colons of ISP-fed mice compared to mice fed dairy-protein based diets (*p* < 0.006). Similarly, alkaline phosphatase concentration, a marker of intestinal cell turnover and immune activation, ranged from 2.28 to 2.99 mg/mL among the dairy-protein fed mice and did not differ between those diet groups (*p* < 0.12). However, small intestinal alkaline phosphatase concentrations in ISP-fed mice (*n =* 5, 4.49 ± 0.82 mg/mL) were higher than those of the collective value of dairy protein-fed mice (*n =* 15, 2.64 ± 0.21 mg/mL, *p* = 0.027). 

### 3.5. Histology

**Figure 1:**
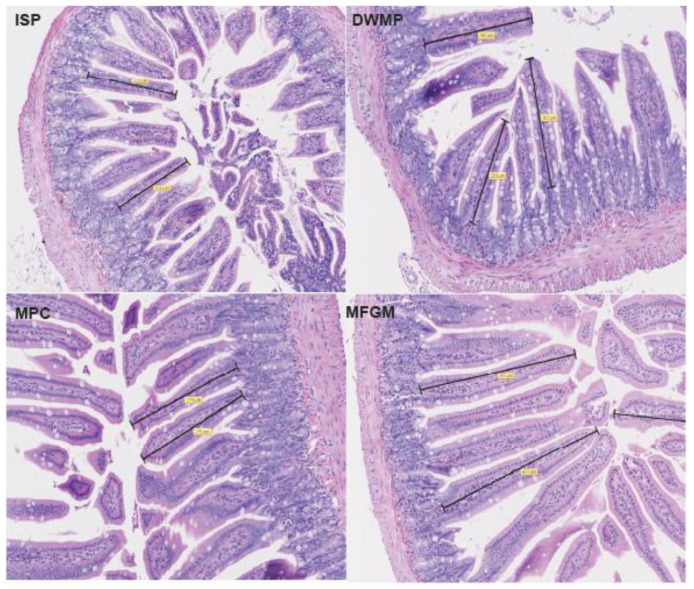
Representative sections from H&E-stained small intestine slides scanned at 20× magnification. Black bars represent distance measured for determination of villi length. ISP, isolated soy protein; DWMP, dried whole milk powder; MPC, milk protein concentrate; MFGM, milk fat globule membrane.

General evaluation of histological sections by a veterinary pathologist showed normal, healthy morphological structures and immunohistochemistry staining for CD3 was not different between diet groups (Dr. Brad Weeks, Texas A&M College of Veterinary Medicine and Biomedical Sciences, personal communication). Villi height and width, crypt depth and width, and smooth muscle thickness measured in H&E-stained distal small intestines (Figure 1) showed that dairy-fed mice had the tallest villi and deepest crypts, although those values did not differ with statistical significance (Table 5).

### 3.6. Gene Expression

**Figure 2:**
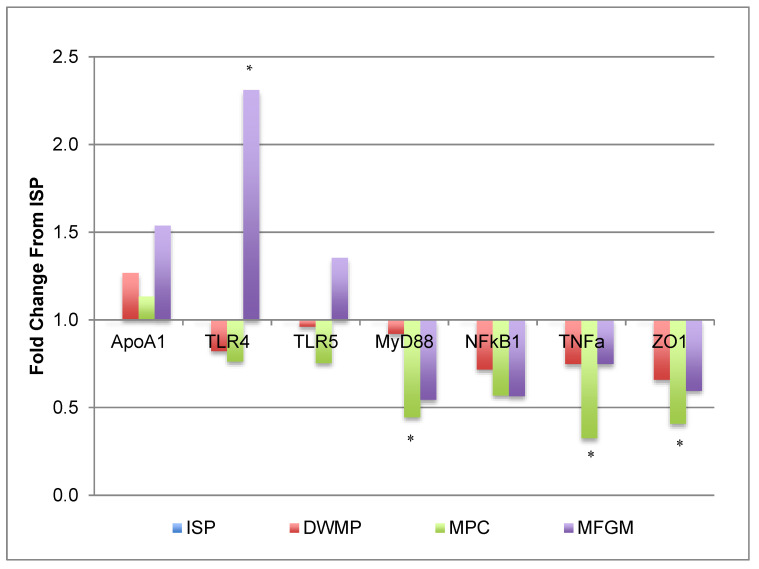
Intestinal gene expression: innate inflammatory markers. Gene expression data were from small intestines of *n* = 5/diet; fold increases were considered significant if at least 2-fold different from the ISP comparator and are denoted by the use of “*” TLR4 was increased 2.3-fold in MFGM compared to ISP; however, downstream responses (*NFkB* and *TNFa*) were modestly downregulated. Tight Junction Protein (*ZO-1*) was downregulated 2.4-fold in MPC compared to ISP.

Fold changes for gene expression in intestinal tissue were generally modest. Marker gene expression related to innate immunity-mediated inflammation, namely *TLR4*, *TLR5*, *MyD88*, *NFκB1*, and *TNFα* showed no indication of overt inflammation in any diet group (Figure 2). MPC showed a distinctive pattern compared to either dairy protein or ISP, the other lard-containing diet. Expression of zona occludin-1, *ZO-1*, also known as Tight Junction Protein 1 (*TJP1*) was similar in mice fed the ISP, DWMP and MFGM diets, while that of MPC-fed mice was 2.4-fold lower than those fed ISP. Likewise, *MyD88* and *TNFα* were reduced in MPC-fed mice relative to intestines of ISP-fed mice. Toll-like receptor 4 (*TLR4*) expression in MFGM-fed mice intestines increased 2.3-fold compared to those fed the ISP diet, however, expression of its downstream genes (i.e., *NFκB* and *TNFα*) were not upregulated. 

**Figure 3:**
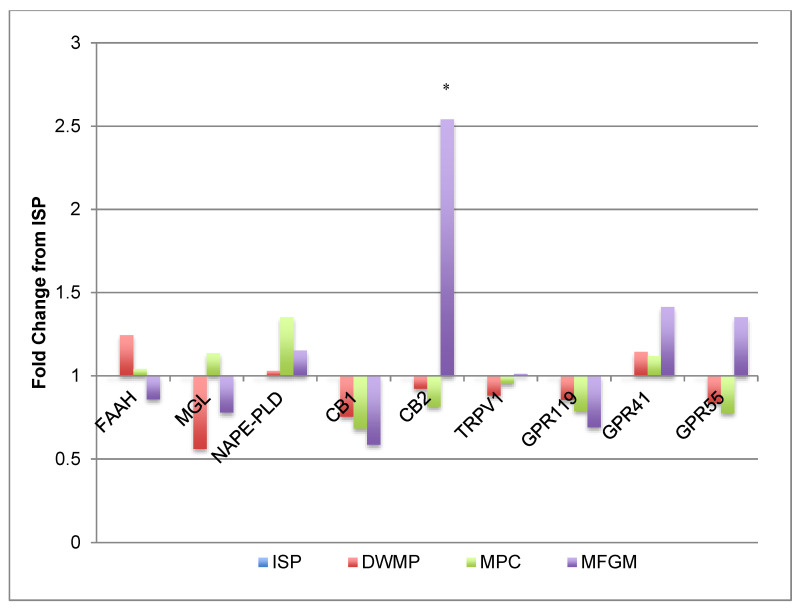
Intestinal gene expression: cannabinoids and endocannabinoids. Gene expression data were from small intestines of *n =* 5/diet; fold increases were considered significant if at least 2-fold different from the ISP comparator and are denoted by the use of “*”. *CB2* was increased 2.5-fold in intestines from milk fat globule membrane (MFGM)-fed mice compared to ISP. Other CB and eCB genes were unchanged.

Intestinal cannabinoid (CB) and endocannabinoid (eCB) gene products play a role in appetite regulation and normal gut physiology [42,43,44,45,46]. As shown in Figure 3, gene expression corresponding to this signaling system did not change with different diets. However, cannabinoid receptor 2 (*CB2*) expression increased 2.5-fold in small intestinal tissue of MFGM-fed mice compared to those of ISP-fed mice. Despite this increase, the expression of downstream CB2-receptor target genes (*TRPV1*, *GPR119*, *GPR41* and *GPR55*) were not different.

**Figure 4:**
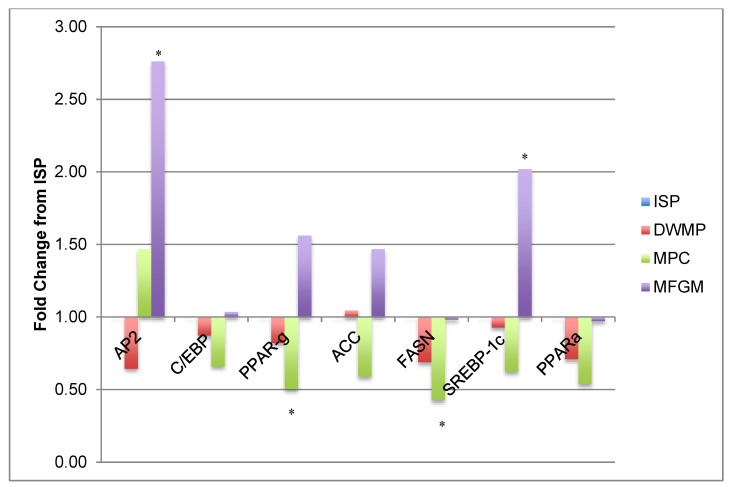
Intestinal gene expression: adipocyte differentiation and lipogenesis. Gene expression data from small intestines of *n =* 5/diet; fold increases were considered significant if at least 2-fold different from the ISP comparator and are denoted by the use of “*”. *AP2* was increased 2.75-fold and *SREBP-1c* increased 2-fold in intestines from MFGM-fed mice compared to ISP. Intestines of milk protein concentrate (MPC)-fed mice showed a 2-fold and 2.3-fold reduction in *PPAR-γ* and *FASN*, respectively, relative to ISP. Other adipocyte differentiation and lipogenesis genes were unchanged.

Figure 4 shows that intestinal *AP2* (adipocyte protein 2, also known as *FABP4*) gene expression was increased 2.76-fold and *SREBP-1c* expression increased 2-fold in MFGM fed mice compared to ISP-fed mice; DWMP- and MPC-fed mice showed similar expression when compared to ISP-fed mice. Conversely, intestinal PPARγ and FASN gene expression in MPC-fed mice was 2-fold and 2.3-fold lower than that of ISP-fed mice, respectively. Expression of these same genes was similar to ISP in DWMP and MFGM. Adipocyte differentiation (*C/EBP*, *PPARγ* and lipogenesis gene (*ACC*) expression were similar among diet treatments. 

### 3.7. Cecal Metabolite Content

Cecal short chain fatty acids and indole concentrations (µM/g cecal content) were similar across all diet groups (Table 6), although butyric acid concentrations were decreased in the ceca of MPC-fed mice (*p* = 0.0003). Relative distributions of SCFA and BCFAs, calculated as both percentage of total and ratios of individual metabolites were also similar among diet groups (data not shown). 

### 3.8. Chow-Fed Reference Mice

**Figure 5:**
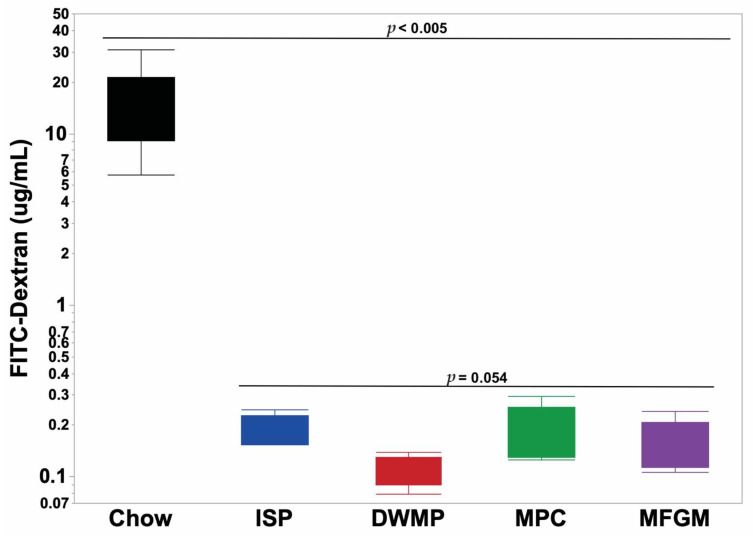
FITC-dextran permeability of chow-fed vs. purified diet-fed mice. Chow-fed mice (*n =* 10) averaged 18.9 ± 5.77 ug/mL FITC-d plasma fluorescence, a 118-fold increase over the average of all purified diet-fed mice. Purified diets (*n =* 60) averaged 0.16 ± 0.01 ug/mL FITC-d plasma fluorescence.

**Figure 6:**
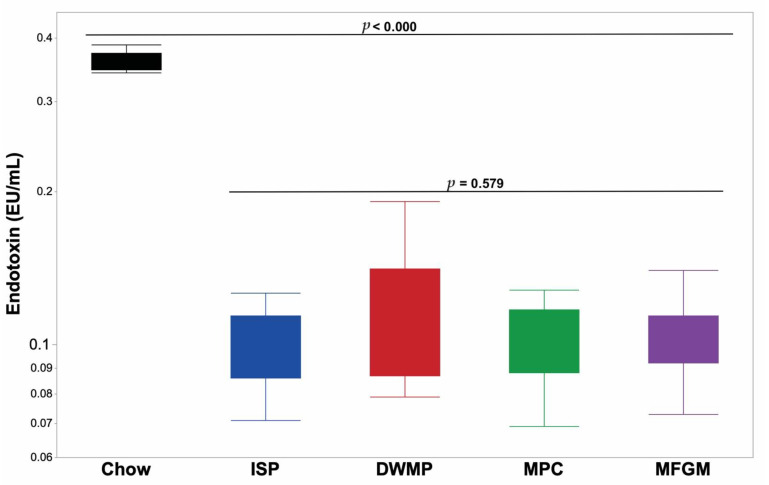
Plasma endotoxin concentrations of chow-fed vs. purified diet-fed mice. Plasma endotoxin content, measured by the limulus amebocyte lysate (LAL) assay, averaged 0.352 ± 0.011 EU/mL for chow-fed mice (*n* = 10) compared to average 0.109 ± 0.004 EU/mL for purified diet-fed mice (*n* = 60). Chow-fed mice showed a 322% increase in plasma compared to the average of purified diet-fed mice.

Mean plasma FITC-dextran fluorescence in chow-fed reference mice (*n =* 10) averaged 18.9 ± 5.8 µg/mL and mean plasma endotoxin concentrations were 0.35 ± 0.01 EU/mL. When compared to PD-fed mice, plasma fluorescence was 95- to 189-fold greater in chow-fed reference mice in conjunction with a 320% increase in endotoxin concentrations (Figure 5; Figure 6). Plasma fluorescence to endotoxin (FITC-dextran/endotoxin) ratios increased 30.8-fold in reference mice compared to the ratio in PD-fed mice (52.0 vs. 1.69, respectively). As with PD-fed mice, plasma cytokine concentrations in chow-fed reference mice were generally low (Supplementary Table S6). However, IL-10 concentrations were 51-fold higher than the average in PD-fed mice (7.24 vs. 0.14 pg/mL). The remaining plasma cytokine concentrations did not differ from those of PD-fed mice or were below the assay’s limit of detection (Supplementary Table S6). 

## 4. Discussion

In general, all PDs produced healthy, immune-competent mice with body weights on the upper limit of established growth curves for C57Bl/6J mice fed nutritionally adequate chow-type diets [47]. Mice consuming the MFGM diet were heavier than mice fed the other diets and gained more weight, resulting in a greater increase in body weight as a percentage of initial body weight. The MFGM diet appeared to favor weight gain through enhanced energy harvest and fat deposition as evidenced by greater feed efficiency and *CB2*, *AP2* and *SREBP-1c* gene expression in the small intestines compared to all other diets [43,44,46]. The increase in feed efficiency did not occur through reduced feed intake due to increased *CB2* gene expression [48] or reduced *PPARα* gene expression. As a result, absolute and fractional weights of liver and RPFPs were greater in MFGM-fed mice compared to those fed MPC or DWMP, and comparable to those of ISP-fed mice. DWMP and MPC fed mice had the lowest average liver and RPFP absolute and fractional weights. MPC-fed mice had the lowest body weights in association with reduced *PPARγ* and *FASN* gene expression. Larger cecal weights in DWMP-fed mice may indicate larger biomass and a higher microbial energy use since cecal weights remained heavier after correcting for final body weight (*p* < 0.010). Conversely, MFGM fed mice had the lowest absolute and fractional cecal weights. Neither dietary fat amount (as g/100 g diet) or degree of dietary fat saturation (%SAT) provided a generic basis to explain the observed differences in weight, adiposity or feed efficiency. All diets had similar amounts of fat, and the %SAT of diets consumed by the two leanest groups, DWMP and MPC, were 58.6% and 35.4%, respectively, identical to those of the two heaviest groups, MFGM and ISP, respectively. 

Previous studies suggested that increased GI-P may allow bacterial inflammogens such as lipopolysaccharide (LPS, or endotoxin) to pass into the systemic circulation and so increase the non-specific inflammatory tone and chronic disease risk [8]. At present, it is unclear whether diet increases “gut leakiness” or whether diet influences microbial production of products such as LPS. This is an important issue, as LPS alone will increase gut permeability [49,50]. Other studies have shown that MFGM improved intestinal integrity and reduced GI-P in LPS-challenged mice [19]. A limitation of the current study was that FITC-dextran exclusion measurements were only performed in one of the three mouse cohorts and this restricted sampling limited the statistical power of our comparisons. Nevertheless, the mean values measured in ISP and MPC-fed mice were equivalent and the highest, with the value for MFGM being 21% lower and that of DWMP-fed mice being 42% lower (*p* < 0.0545). Plasma endotoxin concentrations were obtained for all mice studied and were consistent with FITC-dextran results. The 2.3-fold decrease in *ZO-1* gene expression in the MPC group was not associated with elevated plasma concentrations of FITC-dextran or endotoxin; ZO-1 protein concentrations were not measured. Both FITC-dextran and endotoxin concentrations from mice fed any of the four PDs were several-fold to orders of magnitude lower than those of sex- and age-matched control mice fed a natural ingredient “chow” type diet. Often, purified diets are considered to be greater stressors to physiological systems than laboratory chow. Thus, the degree to which the intestinal integrity of chow-fed animals seemed to be compromised compared to those fed purified diets was an unexpected observation. Specifically designed studies are required to test the effect of diet containing either purified or non-purified ingredients on indicators of intestinal integrity. In poultry, certain cereal grains can increase dietary viscosity and compromise gut integrity [51], but our studies were not designed to evaluate this variable. Nevertheless, the current results support the conclusion that the gastrointestinal integrity of immune-competent mice is not compromised by moderate-fat diets formulated with quality protein sources despite a high SAT% of dietary fat, including that provided by dairy fat.

Whole-fat dairy products may subtly improve intestinal integrity and nutrient sensing as evidenced by decreased GI-P and changes in intestinal *CB2* and *SREBP-1c* gene expression produced by the MFGM-containing diet. In addition to the association with intestinal integrity [52,53], *CB2* is highly associated with regulating innate immune function as a mediator of the immunosuppressive effects of eCBs [52,54]. Others have reported that pretreatment of endothelial cells with CB2 agonists successfully lowers neutrophil activation and reduces TNF-α release [55]. Increased *CB2* expression after MFGM feeding may prime intestinal tissues for improved immune response in a challenge model. Further studies are needed to elicit the mechanism by which dietary dairy fat strengthens intestinal integrity and response to the dietary environment. 

Microbial metabolites, butyrate and indole, are believed to improve intestinal health [6,56]. Notably, butyric acid concentrations were lowest in ceca of MPC-fed mice compared to values from mice fed the other three diets which were higher and similar (*p* < 0.001). A possible reason for this effect is not immediately apparent other than perhaps the loss of water soluble components during protein concentration via ultrafiltration and diafiltration. Cecal indole, a tryptophan metabolite that has been hypothesized to improve tight junction proteins [6] and act as an anti-inflammatory in models of mucosal injury [57,58], did not differ between dietary treatments. Dietary tryptophan contents were similar in all diets (Table S2). While analyzing the ratios of SCFA and BCFAs, we observed a linear relationship between isovalerate and isobutyrate. This linear relationship was also reported by Cardona et al. [59] across four different species and multiple rearing/housing conditions. Tjellström et al. [60] reported a similar relationship in both healthy children and those diagnosed with Celiac disease. Cardona hypothesized that the linear relationship between isobutyrate and isovalerate was not due to dietary effects, but rather due to the shedding of the intestinal epithelium. In our study, there was no significant diet or cohort effect on the ratio of isobutyrate to isovalerate. With the exception of butyrate in MPC-fed mice, microbial metabolites (SCFA/BCFAs and indole) varied little between dietary treatments. It may be that the similarity in macronutrient distribution, carbohydrate sources, and protein quality limited the divergence in possible fermentation products.

Colonic MPO activity is a marker of gut health that showed marked changes with different dietary protein sources. Myeloperoxidase activity, a biomarker of neutrophil infiltration, increased more than 370% in colonic tissues of ISP-fed mice. MPO has been noted to mediate activation of neutrophils, the release of pro-inflammatory cytokines, and to gastrointestinal disease, such as colitis [61,62]. In Fisher-344 rats, MFGM reduced aberrant crypt foci [18], a process associated with increased MPO activity. Studies noting inflammatory reductions with NFDM and MFGM diets did not measure MPO activity [17,19]; given the significant reductions of plasma cytokines and morbidity/mortality rates in those earlier studies, and our observation on increased MPO with ISP feeding, the possibility that dairy proteins influence intestinal neutrophil infiltration and the CB/eCB systems should be explored. 

While intestinal histology was similar among groups there were some observations that merit comment in relation to other literature reports. For example, DWMP-fed mouse intestines had the thickest smooth muscle. Prior studies reported increases in ileal smooth muscle thickness in association with obesity in rats [63]. However, our observed changes cannot be attributed to these causes as DWMP-fed mice were not obese nor did our diets contain varying fiber types, that could contribute to changes in small intestinal smooth muscle thickness [64]. In another consideration, the distal small intestines of MFGM-fed mice had taller, wider villi than both ISP- and DWMP-fed mice. Shorter, wider villi have been associated with disease and impaired nutrient absorption, while certain disease models resulting in lengthened villi are accompanied by a marked loss of goblet cells [65]. MFGM-fed mice showed increased intestinal villi length without loss of goblet cells, potentially providing a larger absorptive surface area consistent with improved feed efficiency. The upregulated *SREPB-1c* gene expression relative to ISP indicates an increased capacity for intestinal lipid in MFGM-fed mouse intestines. Combined with the subtle increase in fat deposition in these mice, it is possible that MFGM primes the intestine for improved nutrient absorption.

Across all immunohistochemistry sections, staining for CD3, a co-receptor of T-lymphocytes, was not grossly different and gave no indication of immune compromise. Likewise, intestinal alkaline phosphatase, associated with mediating epithelial cell damage in the small intestine [66,67], remained unaffected by dietary changes in our study. Thomas et al., previously showed that soy protein, supplemented with Ca^2+^ equivalent to levels found in non-fat dry milk, promoted obesity in mice and adipose tissue inflammation [17]. In non-mammalian models, increased dietary Ca^2+^ provoked naturally occurring necrotic enteritis and was associated with an increased mortality [68]. DWMP contained the highest dietary calcium and calcium-to-phosphorus ratio (Table 1). In our hands, DWMP’s increased dietary calcium did not affect GI-P nor did it promote fat deposition relative to ISP. Furthermore, systemic inflammatory markers (plasma cytokines and IgA), were low and not indicative of an inflammatory state. High-fat diets, where fat provides 45–60% of calories, and DSS-induced colitis models produced plasma cytokines 4–200 times higher than those observed in our mice [35,36,37,69].

IL-1β potentially serves as messenger in inter-organ crosstalk during the development on metabolic disorders, such as insulin resistance [70,71]. IL-1 β concentrations were low and near the limit of detection in ISP-fed mice, while MPC-fed mice had the highest mean plasma concentrations that elevation failed to reach statistical significance due to high variability within the dairy treatments. Despite slight increases in fat pad weights in the MFGM-fed mice, *CD11d* expression in adipose tissue of these mice was not different from the other diet groups. This observation is consistent with the low circulating plasma cytokine concentrations. Importantly, none of the diets in our study resulted in IL-1 β concentrations reaching levels seen in inflammatory models of obesity or colitis [38,39]. Thus, intestinal integrity and systemic inflammatory tone appear to be unaffected by isolated soy protein, whole milk or dairy milk fractions, or varying Ca^2+^ levels in these diets. 

There were differences in cholesterol among the diets; however, no diet included exogenous bile acid or have cholesterol concentrations reach those required to induce adverse events such as atherosclerosis or steatohepatitis in mice. Our diets contained 0.01–0.04% cholesterol, well below the 1.2–5% dietary cholesterol needed in conjunction with 0.5–2% sodium cholate to induce atherosclerotic events in wild-type mice [72]. Additionally, diets containing 1% cholesterol were shown to be insufficient to induce inflammatory mechanisms in diet models of NAFLD and NASH in C57Bl6 mice [73]. Thus, we do not believe that the varying cholesterol contents would affect the phenotypes measured not provide a dietary environment to induce inflammatory events or disease in these mice.

Marked changes were observed in GI-P measures for chow-fed reference mice compared to those fed PDs. Since all reference mice were healthy, the degree of increase in FITC-dextran permeability and plasma endotoxin was highly unexpected. As such, it is difficult to determine whether the differences are due to non-purified diet consumption, housing and transportation stress, or some previously encountered unknown external challenges. Furthermore, this study shows the potential for variation between reared cohorts of mice from the same genetic background and commercial source. It is unlikely that the animals were in an inflamed state as evidenced by physiologically low plasma cytokine concentrations. Given the statistical increase in IL-10 in these mice, it is plausible that appropriate immune surveillance in chow-fed mice prevented excessive activation of the adaptive immune system activation. Thus, the validity of solely relying upon comparisons of FITC-dextran and plasma endotoxin concentrations in studies conducted at different times or different laboratories may be inappropriate. Care should be taken when using chow-fed animals for reference data in nutritional studies.

## 5. Conclusions

Overall, PDs containing isolated soy or dairy proteins with macronutrient distributions meeting recommendations for breeding mice, and consistent with those from the Dietary Guidelines for Americans promoted healthy animal growth and gastrointestinal immune status. Subtle differences could be observed between dietary groups, but these differences largely failed to suggest the presence of disease provoking inflammation or altered gastrointestinal morphology in mature animals. In this context, dairy milk in the form of full-fat milk should be considered a healthy, animal-based protein source. The different directional effects of MPC and MFGM on a number of variables measured here, including bodyweight and gene expression, support the consideration of milk as a highly complex bioactive food whose intact properties provide greater overall benefit. Isolated soy protein effects on colonic MPO activity deserve additional study to determine the mechanism of increase.

Given the marked increase in GI-P and plasma endotoxin of reference chow-fed mice compared to PD-fed mice, it is apparent that such values alone may not provide a proper assessment of animal health status or gastrointestinal function. Therefore, dietary-feeding trials specifically designed to investigate the relationships among diet type/quality and gastrointestinal integrity are required. Specifically, diets with similar macronutrient compositions but varying degrees of carbohydrate purification are needed to evaluate whether highly processed diets, as modeled by PDs, represent fundamental stress on gastrointestinal integrity.

## Figures and Tables

**Table 1:** Diet Composition.

	ISP	DWMP	MPC	MFGM
Protein, % by weight	18.7	18.0	18.6	18.6
Carbohydrate, % by weight	46.9	46.4	47.8	47.1
Fat, % by weight	12.2	12.4	12.1	12.5
Cholesterol mg/kg	95	378	139	74
Cholesterol (% by weight)	0.01	0.04	0.01	0.01
Calcium (%)	0.37	0.89	0.55	0.12
Ca:P	1.37	3.62	1.49	1.67
Protein, % kcal from	20.1	19.5	19.9	19.8
Carbohydrate, % kcal from	50.4	50.3	51.1	50.2
Fat, % kcal from	29.5	30.2	29.1	30
Saturated Fat, % of total fat	33.8	58.6	35.4	55.5
MUFA, % of total fat	39.7	26.5	40.1	31.5
PUFA, % of total fat	26.5	14.9	24.5	12.9
Kcal/g	3.7	3.7	3.7	3.8

Values are calculated from ingredient analysis or manufacturer data. ISP, isolated soy protein; DWMP, dried whole milk powder; MPC, milk protein concentrate; MFGM, milk fat globule membrane; MUFA, monounsaturated fatty acids; PUFA, polyunsaturated fatty acids.

**Table 2:** Animal Growth and Feed Intake.

	ISP	DWMP	MPC	MFGM	*p*-Value
Start Weight	16.0 ± 1.24	16.9 ± 1.24	16.0 ± 1.24	16.7 ± 1.24	0.183
Final Weight	30.0 ± 1.49 ^b^	30.3 ± 1.49 ^b^	29.6 ± 1.49 ^b^	32.3 ± 1.49 ^a^	0.002
Weight Gain	13.7 ± 1.49 ^b^	13.9 ± 1.49 ^b^	13.2 ± 1.49 ^b^	16.0 ± 1.49 ^a^	0.002
Total Feed Disappearance	256 ± 7.09 ^ab^	267 ± 7.09 ^a^	252 ± 7.09 ^b^	263 ± 7.08 ^a^	0.004
Feed Efficiency	19.6 ± 1.89 ^a^	20.3 ± 1.89 ^a^	19.9 ± 1.89 ^a^	17.5 ± 1.89 ^b^	0.004

Data represents values from all cohorts; *n =* 15 mice/diet group. Values are means (g) ± SEM; feed efficiency values are means (g feed/g gain) ± SEM. Different letters (a,b) signify differences between groups (*p* < 0.05). ISP, isolated soy protein; DWMP, dried whole milk powder; MPC, milk protein concentrate; MFGM, milk fat globule membrane.

**Table 3:** Organ Weights.

	ISP	DWMP	MPC	MFGM	*p*-Value
Liver	1.21 ± 0.05 ^ab^	1.10 ± 0.05 ^b^	1.08 ± 0.05 ^b^	1.34 ± 0.05 ^a^	0.000
Cecum	0.32 ± 0.02 ^ab^	0.39 ± 0.02 ^a^	0.31 ± 0.02 ^b^	0.28 ± 0.02 ^b^	0.001
Retroperitoneal Fat Pad	0.33 ± 0.06 ^ab^	0.27 ± 0.06 ^b^	0.30 ± 0.06 ^b^	0.36 ± 0.06 ^a^	0.004
Gastrocnemius Muscle	0.16 ± 0.01	0.16 ± 0.01	0.15 ± 0.01	0.16 ± 0.01	0.292
Spleen	0.081 ± 0.007	0.078 ± 0.008	0.080 ± 0.007	0.083 ± 0.007	0.825
Thymus (mg)	32.3 ± 1.93	32.7 ± 2.09	34.4 ± 1.94	33.2 ± 2.06	0.868

Data represents values from all cohorts; *n =* 15 mice/diet group unless otherwise noted. Values are means (g) ± SEM; thymus values are means (mg) ± standard errors. Different letters (a,b) signify differences between groups (*p* < 0.05). ISP, isolated soy protein; DWMP, dried whole milk powder; MPC, milk protein concentrate; MFGM, milk fat globule membrane.

**Table 4:** Markers of gastrointestinal (GI) integrity and systemic inflammatory tone.

	ISP	DWMP	MPC	MFGM	*p*-value
Transit Time (hours) *n =* 5	3.24 ± 0.37	2.83 ± 0.39	3.14 ± 0.60	3.58 ± 1.42	0.930
Endotoxin (EU/mL) *n =* 15	0.10 ± 0.01	0.11 ± 0.01	0.11 ± 0.01	0.11 ± 0.01	0.875
FITC-Dextran (ug/mL) *n =* 5	0.19 ± 0.02	0.11 ± 0.01	0.19 ± 0.03	0.15 ± 0.02	0.0545
IgA (ug/mL) *n =* 15	161 ± 18.2 ^c^	212 ± 18.2 ^b^	204 ± 18.2 ^bc^	285 ± 18.2 ^a^	0.000
IFN-γ (pg/mL)	<LOD	<LOD	0.04 ± 0.04	<LOD	--
IL-1β (pg/mL)	0.55 ± 0.55	3.59 ± 3.47	5.08 ± 1.75	3.18 ± 1.52	0.483
IL-6 (pg/mL)	100 ± 52.9	41.9 ± 23.5	57.6 ± 18.9	35.4 ± 14.7	0.469
IL-12p70 (pg/mL)	8.81 ± 3.66	7.11 ± 3.55	4.42 ± 1.52	1.84 ± 1.23	0.295
MIP-2 (pg/mL)	112 ± 43	90.8 ± 38	94.6 ± 26	96.0 ± 25	0.971
TNF-α (pg/mL)	<LOD	<LOD	18.2 ± 7.14 ^a^	4.84 ± 1.67 ^b^	0.007
IL-10 (pg/mL)	0.21 ± 0.21	<LOD	<LOD	0.35 ± 0.28	0.438
Eotaxin (pg/mL)	4215 ± 238	4107 ± 172	3773 ± 201	3780 ± 211	0.318
MPO (uU/mL) *n =* 5	36.3 ± 10.7 ^a^	9.75 ± 2.59 ^b^	8.16 ± 1.70 ^b^	8.36 ± 1.23 ^b^	0.006
ALP (mg/mL) *n =* 5	4.49 ± 0.90	2.65 ± 0.38	2.28 ± 0.26	2.99 ± 0.26	0.118

Values are means ± SEM; Different letters signify differences between groups (*p* < 0.05). Myeloperoxidase (MPO) results are expressed as uU/mL, where 1 uU = 1 pmol/min activity. ISP, isolated soy protein; DWMP, dried whole milk powder; MPC, milk protein concentrate; MFGM, milk fat globule membrane.

**Table 5:** Small intestine morphology.

	ISP	DWMP	MPC	MFGM	*p*-Value
Villous Height	234 ± 27.0	264 ± 29.8	269 ± 18.9	262 ± 41.8	0.760
Villous Width	64.0 ± 3.65	58.9 ± 4.55	66.2 ± 1.48	71.0 ± 1.41	0.104
Crypt Depth	75.7 ± 3.63	87.9 ± 4.27	89.9 ± 3.90	86.8 ± 5.89	0.077
Crypt Width	27.8 ± 1.93	27.3 ± 0.86	30.3 ± 0.62	28.9 ± 0.80	0.594
Smooth Muscle	33.5 ± 2.02	40.5 ± 3.74	32.9 ± 2.06	31.2 ± 3.54	0.189

Data represents values from *n =* 5 mice/diet group. Individual data points were averaged to produce a single value per mouse for each measure. Values are means (um) ± SEM. (*p* < 0.05). ISP, isolated soy protein; DWMP, dried whole milk powder; MPC, milk protein concentrate; MFGM, milk fat globule membrane.

**Table 6:** Cecal metabolite content.

	ISP	DWMP	MPC	MFGM	*p*-Value
Indole	159 ± 99.3	239 ± 101	90.4 ± 100	252 ± 97.4	0.274
Acetic Acid	23.8 ± 5.15	17.5 ± 5.14	18.3 ± 5.17	21.7 ± 5.14	0.066
Propionic Acid	1.79 ± 0.42	1.60 ± 0.42	1.67 ± 0.44	2.37 ± 0.42	0.428
Butyric Acid	4.23 ± 0.32 ^a^	3.48 ± 0.32 ^a^	2.21 ± 0.33 ^b^	3.47 ± 0.32 ^a^	0.000
Isobutyric Acid	0.26 ± 0.09	0.22 ± 0.09	0.25 ± 0.10	0.25 ± 0.09	0.836
Isovaleric Acid	0.11 ± 0.04	0.09 ± 0.04	0.08 ± 0.04	0.11 ± 0.04	0.107
Valeric Acid	0.25 ± 0.09	0.23 ± 0.09	0.20 ± 0.09	0.30 ± 0.09	0.129
Total SCFA	30.4 ± 2.93	23.1 ± 2.93	23.2 ± 3.03	28.2 ± 2.93	0.209

Indole values represent concentrations in nMol/g cecal content ± SEM. Short-chain fatty acids (SCFA) values are expressed as average uMol/g cecal content ± SEM. Total SCFAs were calculated by adding the concentrations of all SCFA measured in cecal content. Different letters signify differences between groups (*p* < 0.05). ISP, isolated soy protein; DWMP, dried whole milk powder; MPC, milk protein concentrate; MFGM, milk fat globule membrane.

## Data Availability

The data presented in this study are available by request to authors.

## Supplementary Materials

The following are available online at https://www.mdpi.com/article/10.3390/nu13041251/s1, Figure S1: Normalized C_T_ (ΔC_T_) values for small intestine inflammatory gene expression, Figure S2: Normalized C_T_ (ΔC_T_) values for small intestine cannabinoid gene expression, Figure S3: Normalized C_T_ (ΔC_T_) values for small intestine adipocyte differentiation and lipogenesis gene expression, Table S1: Detailed dietary ingredients for all diets, Table S2: Composed diet analyses: amino acids, Table S3: Composed diet analyses: fatty acid profiles, Table S4: Analyzed gene targets and qPCR primer sequences, Table S5: Organ weights as percent of body weight, Table S6: Plasma cytokine concentrations of chow-fed vs. purified diet-fed mice.
