# Supplementary material for: Whole and Isolated Protein Fractions Differentially Affect Gastrointestinal Integrity Markers in C57Bl/6 Mice Fed Diets with a Moderate-Fat Content
Source: Nutrients. 2021 Apr 10;13(4):1251. doi: 10.3390/nu13041251 (PMC8069602; doi:10.3390/nu13041251)
Supplement: Supplementary file 1 [file nutrients-13-01251-s001.pdf]

Supplementary Table 1. Detailed Dietary Ingredients for All Diets

| **Ingredient (g/kg)** | **ISP** | **DWMP** | **MPC** | **MFGM** |
|---|---|---|---|---|
| Isolated Soy Protein | 210 | | | |
| Dried Whole Milk Powder | | 390 | | |
| Milk Protein Concentrate | | | 230 | |
| Milk Fat Globule Membrane Protein | | | | 325 |
| Casein | | 95 | | |
| DL-Methionine | 2.5 | | | 3 |
| L-Cystine | 1.05 | 2.2 | 2.3 | 1.05 |
| Sucrose | 190 | 40 | 190 | 190 |
| Corn Starch | 188.13 | 175.58 | 189.28 | 178.68 |
| Maltodextrin | 100 | 100 | 100 | 100 |
| Cellulose | 140 | 140 | 140 | 110 |
| Soybean Oil | 20 | 20 | 20 | 20 |
| Anhydrous Milkfat | | | | 27 |
| Lard | 100 | | 95 | |
| Mineral Mix, w/o Ca & P (98057) | 17 | 17 | 17 | 17 |
| Vitamin Mix, AIN-93-VX (94047) | 12.7 | 12.7 | 12.7 | 12.7 |
| Calcium Phosphate, dibasic | 10.9 | | 0.5 | 9.2 |
| Calcium Carbonate | 4.5 | 4.3 | | 3.15 |
| Choline Bitartrate | 3.2 | 3.2 | 3.2 | 3.2 |
| TBHQ, antioxidant | 0.02 | 0.02 | 0.02 | 0.02 |

Supplementary Table 2. Composed Diet Analyses: Amino Acids* (% w/w)

| | *ISP* | *DWMP* | *MPC* | *MFGM* | *CHOW* |
|---|---|---|---|---|---|
| *Taurine* | 0.15 | 0.15 | 0.22 | 0.22 | 0.16 |
| *Hydroxyproline* | 0.00 | 0.00 | 0.14 | 0.00 | 0.01 |
| *Aspartic Acid* | 2.08 | 1.31 | 1.78 | 1.41 | 2.26 |
| *Threonine* | 0.68 | 0.74 | 1.13 | 0.81 | 0.89 |
| *Serine* | 0.82 | 0.80 | 0.82 | 0.92 | 0.98 |
| *Glutamic Acid* | 3.64 | 3.87 | 2.87 | 3.96 | 4.17 |
| *Proline* | 0.92 | 1.72 | 0.87 | 1.76 | 1.34 |
| *Lanthionine* | 0.00 | 0.00 | 0.00 | 0.00 | 0.00 |
| *Glycine* | 0.79 | 0.36 | 0.43 | 0.35 | 1.23 |
| *Alanine* | 0.81 | 0.58 | 0.87 | 0.62 | 1.21 |
| *Cysteine* | 0.32 | 0.26 | 0.46 | 0.31 | 0.38 |
| *Valine* | 0.95 | 1.17 | 1.01 | 1.21 | 1.23 |
| *Methionine* | 0.48 | 0.44 | 0.62 | 0.52 | 0.38 |
| *Isoleucine* | 0.92 | 0.96 | 1.03 | 0.99 | 1.05 |
| *Leucine* | 1.52 | 1.72 | 1.81 | 1.84 | 1.91 |
| *Tyrosine* | 0.58 | 0.78 | 0.42 | 0.77 | 0.72 |
| *Phenylalanine* | 1.01 | 0.90 | 0.64 | 0.91 | 1.15 |
| *Hydroxylysine* | 0.02 | 0.03 | 0.02 | 0.02 | 0.05 |
| *Ornithine* | 0.01 | 0.00 | 0.00 | 0.00 | 0.02 |
| *Lysine* | 1.18 | 1.42 | 1.62 | 1.52 | 1.40 |
| *Histidine* | 0.47 | 0.50 | 0.36 | 0.52 | 0.59 |
| *Arginine* | 1.34 | 0.62 | 0.54 | 0.64 | 1.55 |
| *Tryptophan* | 0.28 | 0.28 | 0.34 | 0.26 | 0.31 |
| **TOTAL** | **18.97** | **18.61** | **18.00** | **19.56** | **22.99** |
| ***Branched Chain Amino Acids*** | **3.39** | **3.85** | **3.85** | **4.04** | **4.19** |

*Dietary amino acid analysis was conducted by the Agricultural Experiment Station Chemical Laboratories at the University of Missouri-Columbia.

Supplementary Table 3. Composed Diet Analyses: Fatty Acid Profiles* (% AUC)

| | *ISP* | *DWMP* | *MPC* | *MFGM* | *CHOW* |
|---|---|---|---|---|---|
| *C6:0* | 0.00 | 0.06 | 0.00 | 0.15 | 0.00 |
| *C8:0* | 0.00 | 0.61 | 0.00 | 0.60 | 0.00 |
| *C10:0* | 0.07 | 1.28 | 0.12 | 2.26 | 0.04 |
| *C11:0* | 0.00 | 0.03 | 0.00 | 0.00 | 0.00 |
| *C12:0* | 0.08 | 2.94 | 0.14 | 2.85 | 0.06 |
| *C13:0* | 0.00 | 0.11 | 0.00 | 0.11 | 0.00 |
| *C14:0* | 1.21 | 9.86 | 1.54 | 9.25 | 0.83 |
| *C14:1* | 0.03 | 1.03 | 0.09 | 0.87 | 0.04 |
| *C16:0* | 21.5 | 29.9 | 22.2 | 27.7 | 15.2 |
| *C16:1* | 1.65 | 1.21 | 1.79 | 1.10 | 1.47 |
| *C18:0* | 10.6 | 11.3 | 11.3 | 12.4 | 3.7 |
| *c18:1t9* | 0.28 | 1.34 | 0.49 | 0.00 | 0.00 |
| *C18:1c9* | 31.9 | 22.2 | 32.0 | 25.5 | 20.4 |
| *C18:1c11* | 4.85 | 3.00 | 5.65 | 3.96 | 3.51 |
| *C18:2* | 24.3 | 12.8 | 21.6 | 11.3 | 46.9 |
| *C18:3* | 2.00 | 1.76 | 1.69 | 1.36 | 5.31 |
| *C20:0* | 0.19 | 0.22 | 0.20 | 0.20 | 0.17 |
| *C20:1* | 0.04 | 0.02 | 0.03 | 0.07 | 0.10 |
| *C20:2* | 0.57 | 0.08 | 0.55 | 0.10 | 0.34 |
| *C20:3* | 0.20 | 0.15 | 0.24 | 0.17 | 0.20 |
| *C20:4* | 0.51 | 0.00 | 0.46 | 0.00 | 0.00 |
| *C22:0* | 0.04 | 0.00 | 0.00 | 0.00 | 0.00 |
| *C22:1* | 0.00 | 0.00 | 0.00 | 0.00 | 0.85 |
| *C22:6* | 0.00 | 0.00 | 0.00 | 0.00 | 0.68 |
| *C24:0* | 0.00 | 0.02 | 0.00 | 0.00 | 0.10 |
| *C24:1* | 0.00 | 0.00 | 0.00 | 0.00 | 0.12 |
| *SFA* | 33.7 | 56.4 | 35.5 | 55.5 | 20.0 |
| *SC SFA* | 1.36 | 14.89 | 1.8 | 15.22 | 0.93 |
| *MUFA* | 39.7 | 26.5 | 40.1 | 31.5 | 26.5 |
| *PUFA* | 26.5 | 14.9 | 24.5 | 12.9 | 53.4 |

*Lipids were extracted from composed diet pellets and fatty acids analyzed by gas chromatography as previously described (Meat Science. 2006;73:432-441).

Supplementary Table 4. Analyzed Gene Targets and qPCR Primer Sequences

| **Gene Name** | **Gene Shortcode** | **Classification** | **FWD Primer Sequence** |
|---|---|---|---|
| B-Actin | *ACTB* | PCR Reference Gene | GGC TGT ATT CCC CTC CAT CG |
| Apolipoprotein A1 | *APOA1* | Inflammation | GCA CGT ATG GCA GCA AGA TG |
| Toll-like receptor 4 | *TLR4* | Inflammation | AGG AAG TTT CTC TGG ACT AAC AAG TTT AG |
| Toll-like receptor 5 | *TLR5* | Inflammation | GCC ACA TCA TTT CCA CTC CT |
| Myeloid differentiation primary response protein 88 | *MYD88* | Inflammation | CTA GGA CAA ACG CCG GAA CT |
| Nuclear factor of kappa light polypeptide gene enhancer in B-cells 1 | *NFκβ1* | Inflammation | ACA CGA GGC TAC AAC TCT GC |
| Tumor necrosis factor - alpha | *TNFα* | Inflammation | CCA CCA CGC TCT TCT GTC TAC |
| Zona occludens protein 1 (aka Tight junction protein 1: TJP1) | *ZO1* | Gut permeability | TTT TTG ACA GGG GGA GTG G |
| Fatty acid amide hydrolase | *FAAH* | eCB-Metabolism | ACA GGC AGG CCT ATA CCC TT |
| Monoacylglycerol lipase | *MGL* | eCB-Metabolism | CAG AGA GGC CAA CCT ACT TTT C |
| N-acylphosphatidylethanolamine phosphlipase D | *NAPE-PLD* | eCB-Metabolism | GGG CGG CTC TCA CTT TCT A |
| Cannabinoid receptor type 1 | *CB1* | eCB-Signaling | CTG ATG TTC TGG ATC GGA GTC |
| Cannabinoid receptor type 2 | *CB2* | eCB-Signaling | TGA CAA ATG ACA CCC AGT CTT CT |
| Transient receptor potential cation channel, subfamily V, member 1 | *TRPV1* | eCB-Signaling | CCT GCA TTG ACA CCT GTG |
| G protein-coupled receptor 119 | *GPR119* | eCB-Signaling | GCC TTC GGA TGG CAT TTG TC |
| G protein-coupled receptor 41 | *GPR41* | eCB-Signaling | TTT CTG AGC GTG GCC TAT CC |
| G protein-coupled Receptor 55 | *GPR55* | eCB-Signaling | CTA TCT ACA TGA TCA ACT TGG CTG TTT |

Supplementary Table 5 (cont.) Analyzed Gene Targets and qPCR Primer Sequences

| | | | |
|---|---|---|---|
| Acetyl-CoA carboxylase | *ACC* | Lipogenesis | TGT TGA GAC GCT GGT TTG TAG AA |
| Fatty acid synthase | *FASN* | Lipogenesis | TTC CAA GAC GAA AAT GAT GC |
| Sterol regulatory element-binding protein 1c | *SREBP-1c* | Lipogenesis | GAT CAA AGA GGA GCC AGT GC |
| Adipocyte protein 2<br>(aka fatty acid binding protein 4: FABP4) | *AP2* | Adipocyte Differentiation | GAT GCC TTT GTG GGA ACC TG |
| CCAAT/Enhancer Binding Protein | *C/EBP* | Adipocyte Differentiation | GAG CCG AGA TAA AGC CAA ACA |
| Peroxisome proliferator-activated receptor gamma | *PPARγ* | Adipocyte Differentiation | CTG CTC AAG TAT GGT GTC CAT GA |
| Peroxisome proliferator-activated receptor alpha | *PPARα* | Adipocyte Differentiation | TCG GCG AAC TAT TCG GCT G |

Supplementary Table 5. Organ Weights as Percent of Body Weight

| | *ISP* | *DWMP* | *MPC* | *MFGM* | *p-value* |
|---|---|---|---|---|---|
| *Liver* | 3.99 ± 0.07[a,b] | 3.67 ± 0.14[b] | 3.62 ± 0.10[b] | 4.13 ± 0.11[a] | 0.003 |
| *Cecum* | 1.09 ± 0.08[a,b] | 1.28 ± 0.11[a] | 1.11 ± 0.04[a,b] | 0.87 ± 0.05[b] | 0.001 |
| *Retroperitoneal Fat Pad* | 1.06 ± 0.07[a,b] | 0.90 ± 0.07[b] | 0.93 ± 0.04[a,b] | 1.14 ± 0.06[a] | 0.024 |
| *Gastrocnemius Muscle* | 0.50 ± 0.05 | 0.53 ± 0.02 | 0.54 ± 0.01 | 0.51 ± 0.02 | 0.701 |
| *Spleen* | 0.35 ± 0.07 | 0.24 ± 0.02 | 0.25 ± 0.01 | 0.27 ± 0.03 | 0.226 |
| *Thymus* | 0.11 ± 0.01 | 0.10 ± 0.01 | 0.11 ± 0.01 | 0.11 ± 0.01 | 0.817 |

Values are means (%BW) ± standard errors; different letters signify differences between groups ($p<0.05$).

Supplementary Table 6. Plasma Cytokine Concentrations of Chow-fed vs Purified Diet-fed Mice

| | ISP | DWMP | MPC | MFGM | Chow |
|---|---|---|---|---|---|
| **IFN-γ** | <LOD | <LOD | 0.04 ± 0.04 | <LOD | <LOD |
| **IL-1β** | 0.55 ± 0.55 | 3.59 ± 3.59 | 5.08 ± 1.75 | 3.18 ± 1.52 | 5.12 ± 1.97 |
| **IL-6** | 100 ± 52.9 | 41.9 ± 24.3 | 57.6 ± 18.9 | 35.4 ± 14.7 | 36.3 ± 26.8 |
| **IL-12p70** | 8.81 ± 3.66 | 7.11 ± 3.67 | 4.42 ± 1.52 | 1.84 ± 1.23 | 12.4 ± 2.29 |
| **MIP-2** | 112 ± 43.0 | 90.8 ± 39.1 | 94.6 ± 25.8 | 96.9 ± 25.2 | 166 ± 23.2 |
| **TNF-α** | 0.43 ± 0.33 | 1.84 ± 1.84 | 18.2 ± 7.14 | 4.84 ± 1.67 | 0.29 ± 0.29 |
| **IL-10** | 0.21± 0.21 | <LOD | <LOD | 0.35 ± 0.28 | 7.24 ± 2.46 |

Values are means (pg/mL) ± standard errors; chow-fed mice data are provided for reference purposes only and were not included in any statistical analyses.

Supplementary Figure 1. Normalized $C_T$ ($\Delta C_T$) values for small intestine inflammatory gene expression

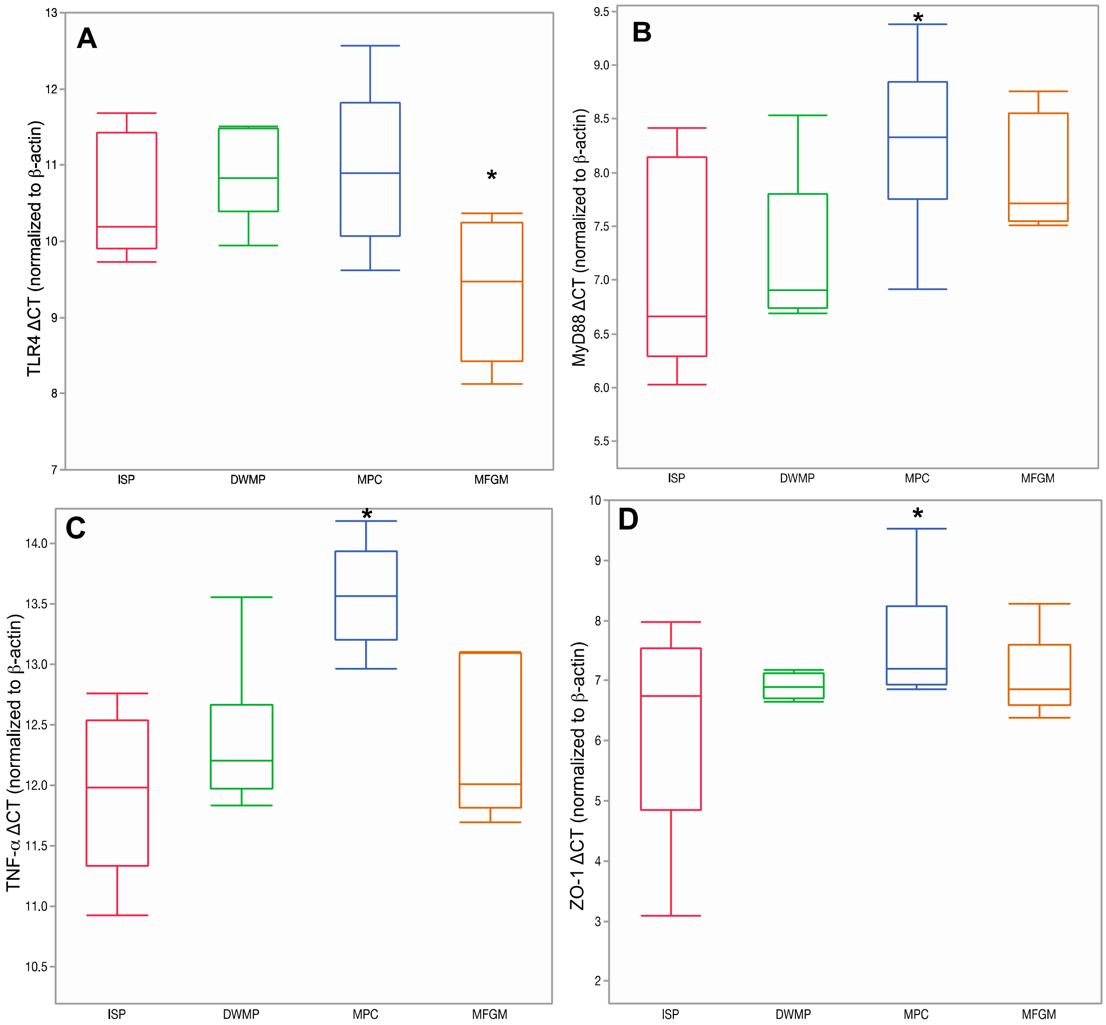


Normalized gene expression values ($\Delta C_T$) are shown for those genes that reached significance of p-value <0.05 ANOVA and Tukey's HSD post-hoc analysis.

Panel A: *TLR4* $\Delta C_T$ was significantly reduced in MFGM compared the other diets ($p = 0.0285$).

Panel B: *MyD88* $\Delta C_T$ was highest in MPC and MFGM, nearing statistical significance ($p = 0.0591$). Panel C: *TNFα* $\Delta C_T$ was highest in MPC ($p = 0.0017$). Panel D: *ZO-1* did not show a difference statistically in $\Delta C_T$ ($p = 0.308$).

Supplementary Figure 2. Normalized $C_T$ ($\Delta C_T$) values for small intestine cannabinoid gene expression

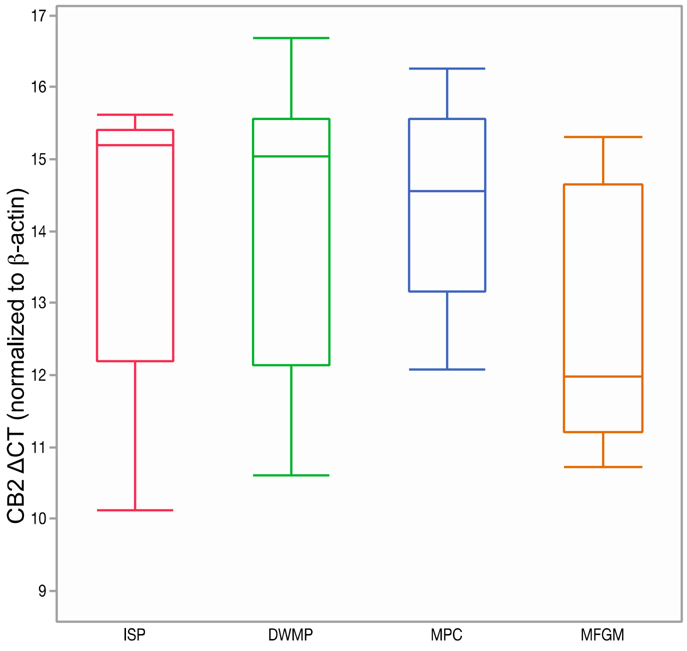


Normalized gene expression values ($\Delta C_T$) are shown for those genes that reached significance by fold-change of greater than 2.0 or less than 0.5 as defined in methods. *CB2* did not show statistical differences for $\Delta C_T$ values ($p = 0.527$), but fold change of MFGM relative to ISP were greater than 2-fold.

Supplementary Figure 3. Normalized $C_T$ ($\Delta C_T$) values for small intestine adipocyte differentiation and lipogenesis gene expression

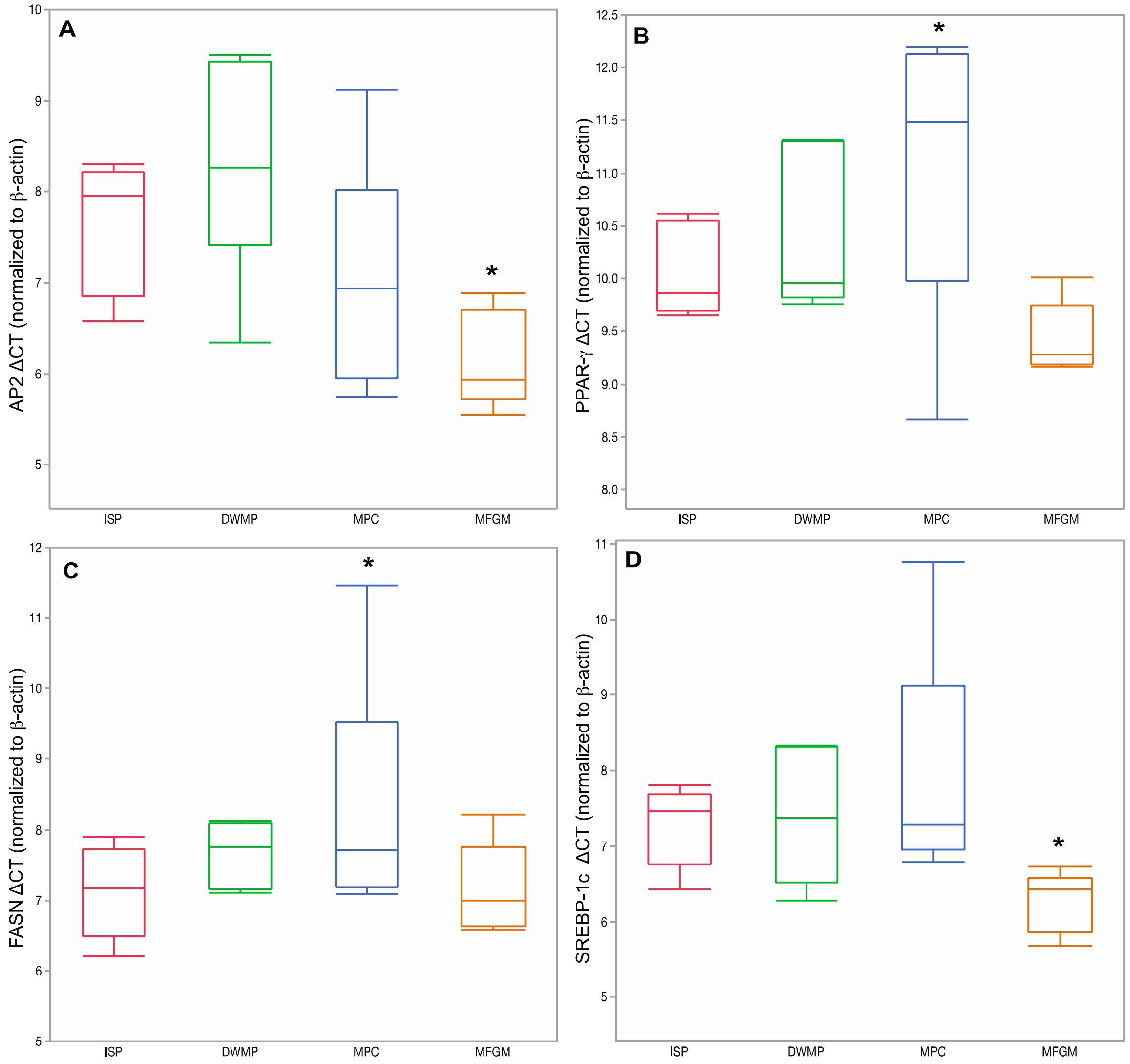


Normalized gene expression values ($\Delta C_T$) are shown for those genes that reached significance by fold-change of greater than 2.0 or less than 0.5 as defined in methods. P-values from ANOVA and Tukey's HSD post-hoc analysis are provided.

Panel A: *AP2* was reduced in MFGM compared the other diets ($p = 0.0208$). Panel B: *PPAR-$\gamma$* was reduced in MFGM compared to DWMP ($p = 0.039$). Panel C: *FASN* did not show a difference statistically in $\Delta C_T$ ($p = 0.179$). Panel D: *SREBP-1c* did not show a difference statistically in $\Delta C_T$ ($p = 0.076$).
